# A multidisciplinary approach to inherited retinal dystrophies from diagnosis to initial care: a narrative review with inputs from clinical practice

**DOI:** 10.1186/s13023-023-02798-z

**Published:** 2023-07-31

**Authors:** Vittoria Murro, Sandro Banfi, Francesco Testa, Giancarlo Iarossi, Benedetto Falsini, Andrea Sodi, Sabrina Signorini, Achille Iolascon, Roberta Russo, Dario Pasquale Mucciolo, Roberto Caputo, Giacomo Maria Bacci, Sara Bargiacchi, Simona Turco, Stefania Fortini, Francesca Simonelli

**Affiliations:** 1 Department of Neuroscience, Psychology, Drug Research and Child Health, University of Florence, Florence, Italy; 2 Eye Clinic, Careggi Teaching Hospital, Florence, Italy; 3 Telethon Institute of Genetics and Medicine (TIGEM), Pozzuoli, NA Italy; 4 Medical Genetics, Department of Precision Medicine, University of Campania Luigi Vanvitelli, Naples, Italy; 5 Eye Clinic, Multidisciplinary Department of Medical, Surgical and Dental Sciences, University of Campania Luigi Vanvitelli, Naples, Italy; 6 Department of Ophthalmology, Bambino Gesù Children’s Hospital, Rome, Italy; 7 Ophthalmology Unit, Fondazione Policlinico Universitario A. Gemelli IRCCS, Rome, Italy; 8 Center of Child Neuro-Ophthalmology, IRCCS, Mondino Foundation, Pavia, Italy; 9 Medical Genetics Unit, Azienda Ospedaliera Universitaria Federico II, Naples, Italy; 10 Department of Molecular Medicine and Medical Biotechnologies, University of Naples Federico II, Naples, Italy; 11 CEINGE-Biotecnologie Avanzate, Naples, Italy; 12 Ophthalmology Unit, San Jacopo Hospital, Pistoia, Italy; 13 Pediatric Ophthalmology Unit, A. Meyer Children’s Hospital IRCCS, Florence, Italy; 14 Medical Genetics Unit, Ospedale Pediatrico Meyer, Florence, Italy; 15 National Centre of Services and Research for the Prevention of Blindness and Rehabilitation of the Visually Impaired, International Agency for the Prevention of Blindness-IAPB Italy Onlus, Fondazione Policlinico Universitario A. Gemelli IRCCS, Rome, Italy

**Keywords:** Inherited retinal diseases, Visual function, Clinical diagnosis, Molecular diagnosis, Multidisciplinary, Early-onset retinal dystrophy, Leber congenital amaurosis, Retinitis pigmentosa

## Abstract

**Background:**

Non-syndromic inherited retinal dystrophies (IRDs) such as retinitis pigmentosa or Leber congenital amaurosis generally manifest between early childhood and late adolescence, imposing profound long-term impacts as a result of vision impairment or blindness. IRDs are highly heterogeneous, with often overlapping symptoms among different IRDs, and achieving a definite diagnosis is challenging. This narrative review provides a clinical overview of the non-syndromic generalized photoreceptor dystrophies, particularly retinitis pigmentosa and Leber congenital amaurosis. The clinical investigations and genetic testing needed to establish a diagnosis are outlined, and current management approaches are discussed, focusing on the importance of the involvement of an interdisciplinary team from diagnosis and initial care to long-term follow-up and support.

**Results:**

The effective management of IRDs requires a multidisciplinary, and ideally interdisciplinary, team of experts knowledgeable about IRDs, with experienced professionals from fields as diverse as ophthalmology, neuropsychiatry, psychology, neurology, genetics, orthoptics, developmental therapy, typhlology, occupational therapy, otolaryngology, and orientation and mobility specialties. Accurate clinical diagnosis encompasses a range of objective and subjective assessments as a prerequisite for the genetic testing essential in establishing an accurate diagnosis necessary for the effective management of IRDs, particularly in the era of gene therapies. Improvements in genome sequencing techniques, such as next-generation sequencing, have greatly facilitated the complex process of determining IRD-causing gene variants and establishing a molecular diagnosis. Genetic counseling is essential to help the individual and their family understand the condition, the potential risk for offspring, and the implications of a diagnosis on visual prognosis and treatment options. Psychological support for patients and caregivers is important at all stages of diagnosis, care, and rehabilitation and is an essential part of the multidisciplinary approach to managing IRDs. Effective communication throughout is essential, and the patient and caregivers’ needs and expectations must be acknowledged and discussed.

**Conclusion:**

As IRDs can present at an early age, clinicians need to be aware of the clinical signs suggesting visual impairment and follow up with multidisciplinary support for timely diagnoses to facilitate appropriate therapeutic or rehabilitation intervention to minimize vision loss.

## Introduction

Non-syndromic inherited retinal dystrophies (inherited retinal diseases; IRDs) form a large family of rare diseases characterized by the progressive dysfunction and loss of photoreceptors and retinal pigment epithelium (RPE), leading to severe impairment of vision or blindness [1–4]. The patterns of inheritance can be autosomal recessive, autosomal dominant, X-linked, or even mitochondrial [2, 5].

Two generalized photoreceptor dystrophies are more likely to be encountered by pediatricians and ophthalmologists in a routine clinical setting: retinitis pigmentosa (RP), the most common IRD [6], and Leber congenital amaurosis (LCA), which is one of the most severe forms [7]. The onset of these conditions is usually between early childhood and late adolescence [3, 8–10].

Due to disease heterogeneity and the overlap of manifestations among different IRDs, achieving a definite clinical diagnosis is often difficult and requires a multidisciplinary effort combining several different assessment techniques [7, 11]. Determining IRD-causing gene variants has also proven complex. However, recent improvements in genome sequencing techniques (notably, the introduction of next-generation sequencing, NGS) have considerably advanced the molecular diagnosis of IRDs [10, 12, 13]. Consequently, establishing the genotype of IRDs is increasingly considered an essential component of the diagnostic workup [8]. The approval in 2017 of voretigene neparvovec, a gene therapy indicated for the treatment of adult and pediatric patients with “vision loss due to inherited retinal dystrophy caused by confirmed biallelic *RPE65* mutations” [14, 15], has raised great expectations and provided further support for the need for molecular diagnosis [12, 16, 17].

The field of IRDs is rapidly evolving, as other gene therapies are under development [1, 18]. These changes pose new challenges to clinicians treating patients with IRDs, including pediatricians, child neuropsychiatrists, and general ophthalmologists. It has become apparent that best-practice care for patients necessitates the involvement of a broad multidisciplinary team experienced in the management of IRDs and including specialists in retinal diseases, the genetics of hereditary retinal dystrophies, vitreoretinal surgery, molecular biology, genetic counseling, patient support, and other technical and bioinformatic aspects of IRDs. In fact, the ideal approach should go beyond the multidisciplinary approach, in which team members work independently on a common issue within defined role responsibilities, to an interdisciplinary one, where teams made up of various disciplines work collaboratively towards a shared, patient-centered outcome, with each discipline building on the expertise of the others.

This narrative review aims to provides a clinical overview of the non-syndromic generalized photoreceptor dystrophies, in particular RP and LCA. The clinical investigations and genetic testing needed to establish a confirmed diagnosis will be outlined, and current management approaches will be discussed, focusing on the importance of the involvement of an interdisciplinary team from diagnosis and initial care through to long-term follow-up and support.

### Clinical overview

Retinitis pigmentosa and LCA are the two IRDs more commonly encountered in clinical practice. Accurate clinical diagnosis of RP and LCA, as with other IRDs, remains a key step in determining the etiopathogenesis of IRDs and effectively managing these conditions. IRDs are highly heterogeneous in phenotypes and genotypes [2, 19–21], and their diagnosis is complicated by the diversity of clinical presentations caused by mutations in a single gene or overlapping clinical phenotypes that can be associated with mutations in different causative genes. Therefore, it is recommended that the clinical diagnosis is refined as much as possible using multiple assessment tools. Patients and parents will have expectations that a definitive diagnosis will be established and accurate information offered on prognosis, risk of genetic transmission within the family, eligibility for treatment, or admission to ongoing clinical trials.

### Retinitis pigmentosa

Retinitis pigmentosa is the most common IRD, with a worldwide prevalence of approximately 1:4000 [6, 22]. The term describes a heterogeneous group of progressive retinal dystrophies characterized by a primary degeneration of rod photoreceptors that progresses to the loss of cone photoreceptors. Often a loss of night vision will be the first symptom the patient notices, followed by progressive concentric reduction of the visual field, ultimately resulting in central vision loss. However, there is usually relative preservation of macula function until an advanced stage of the disease [6, 23]. The majority of forms of RP are associated with mutations of a single gene and display autosomal dominant, autosomal recessive, X-linked or, more rarely, mitochondrial patterns of inheritance [5, 6]. Patients with X-linked RP, representing between 5 and 15% of patients with RP, tend to have a more severe disease course than those with autosomal recessive RP (50–60% of RP patients) [6]. The remaining 30–40% of RP patients have an autosomal dominant form, which is associated with a better outcome in terms of retaining central vision [6].

Although the rhodopsin (*RHO*) gene was the first gene implicated in autosomal RP, the involvement of mutations in over 100 genes has since been identified [24], and genetic overlap with other IRDs further complicates the classification of RP [6]. The high number of genetic mutations associated with RP accounts for the wide heterogeneity exhibited by patients with the disease, with additional implicated genes progressively added as research into RP continues. However, *RHO* remains the gene most associated with autosomal dominant RP, accounting for more than 30% of cases, followed by *PRPH2*, *PRPF31*, and *RP1* [6].

Fundus abnormalities usually affect both eyes and are symmetrical; they typically show bone spicule pigmentation, occurring mainly in the periphery (and/or mid-periphery), narrowing of the retinal vessels, and a waxy optic nerve head pallor. These signs are hallmarks of the condition [6, 25]. During the early stages of RP, when bone spicule pigment deposition is absent or sparse, vascular attenuation is minimal, and the appearance of the optic disc is normal, the fundus may appear normal on examination. Additionally, before the appearance of the typical abnormalities of RP, nonspecific abnormalities such as vitreous changes (cellophane aspect due to increased reflexes from the internal limiting membrane, broadening of the foveal reflex, vitreous condensation), and small, discrete, pale-colored local lesions may be present at the level of the RPE in some patients. Furthermore, the bone spicules at the fundus typical of RP do not develop in all patients, and the degree of hyperpigmentation can vary among patients. Macular complications may develop in RP, including epiretinal membrane formation, macular hole, and cystoid macular edema (CME), reported in up to 50% of patients [26].

The progression of RP is undertaken using visual field analysis, visual acuity, fundus autofluorescence (FAF) photography, and optical coherence tomography (OCT). Multimodal imaging is increasingly being used to monitor the progression and severity of the disease. Several potential biomarkers have been identified, including the width of the ellipsoid zone retinal thinning on OCT or the diameter of the perifoveal hyperautofluorescent ring [27–30].

### Leber congenital amaurosis

Leber congenital amaurosis represents the most severe form of IRD in infancy. It is typically characterized by an early onset within the first months of life and from as early as six weeks after birth with a profound visual deficit, absence of fixation, and a range of other signs and symptoms, including photophobia, nyctalopia, nystagmus, roving eye movements, and the oculodigital sign, together with a highly variable appearance of the fundus [7, 31, 32]. A milder form, namely early-onset severe retinal dystrophy/severe early childhood onset retinal dystrophy (EOSRD/SECORD), usually presents after infancy and within the first five years of age, with a less severe impact on visual acuity and relatively more preserved full field electroretinogram (ffERG) responses according to different genotype–phenotype correlations [32]. Notably, despite a significant overlap of causative genes for the two phenotypes, mutations in some genes like *GUCY2D*, *AIPL1,* and *CEP290* are more frequently associated with LCA, whereas others such as *RPE65*, *LRAT,* and *RDH12* are commonly associated with EOSRD [33]. The ethnic background of patients may influence the frequency of mutations, with mutations in *CEP290*, *GUCY2D*, and *RPE65* showing a prevalence of approximately 10% or higher, generally more frequent in Caucasian populations [33–35]. Conversely, *CRB1* mutations, associated with 13.6% of LCA cases, have been reported as the leading causative genetic defect in the Chinese population, followed by mutations in *GUCY2D* [36]. LCA represents approximately 5% of all IRDs, with a prevalence ranging from 1/30,000 to 1/81,000 [7].

The most common refractive error is hyperopia, particularly when associated with mutations in *GUCY2D;* high myopia has also been reported suggesting an impaired process of emmetropization [31]. Associated ocular features are keratoconus and juvenile cataracts [7]. Gene mutation-dependent renal and olfactory dysfunction have also been reported [31]. Fundus appearance presents high variability ranging from normal to severely disrupted retinal architecture that may include peripheral pigmentary retinopathy or macular atrophy [7]. Electrophysiological tests may be key in assessing patients with suspected LCA presenting nystagmus and apparent normal fundus early in life; ffERG responses are usually undetectable or severely reduced, although a residual cone response has been reported in *GUCY2D* mutation and a residual rod response in *RPE65* [37]. The improvements in retinal imaging technologies like spectral domain OCT (SD-OCT) have revealed the specific retinal structural alterations associated with a particular genotype, allowing a better phenotype-genotype characterization.

In specific genetic mutations, such as *GUCY2D*, *CEP290,* and *RPE65*, the central foveal structure may be relatively preserved, which paves the way to potential therapeutic strategies for genetic remodeling. The identified genes account for about three-quarters of LCA cases, and recent data from the Retinal Information Network lists 26 genes associated with LCA [24]. The majority of causative mutations in LCA have an autosomal recessive pattern of inheritance (except for *CRX*, *IMPDH1*, and *OTX2*) and can affect all of the molecular mechanisms involved in vision: phototransduction (*GUCY2D*, *AIPL1*, *RD3*, *KCNJ13*), retinoid cycle (*RPE65*, *LRAT*, *RDH12*), ciliary transportation (*LCA5*, *CEP290*, *RPGRIP1*, *SPATA7*, *TULP1*, *IQCB1*), photoreceptor morphogenesis (*CRX*, *CRB1*, *GDF6*, *PRPH2*), guanine synthesis (*IMPDH1*), and photoreceptor differentiation (*OTX2*) [37]. However, the underlying mechanism implicated in the LCA phenotype of the recently-identified mutations in *USP45* (LCA 19) and other genes remains to be clarified [38].

## Multidisciplinary care of patients with inherited retinal dystrophies

### Overview

When a suspicion of an IRD is identified, the initial comprehensive neurological, ophthalmologic, and perception tests to identify the patient’s clinical profile will encompass the development profile of the child together with the etiology of the visual disability, functional vision and also neuropsychiatric aspects such as cognition, learning, and socio-emotional aspects. The etiopathogenetic diagnosis of the visual deficit begins the evaluation of any associated neurological or systemic symptoms related to syndromic forms of retinal dystrophy and is a prerequisite for genetic testing.

An accurate determination of the patient’s visual function is critical, and objective and subjective measures are used in combination. The diagnostic process should involve an accurate medical history, with inquiries about the existence of other family members with visual impairment or systemic diseases (e.g., hearing loss, kidney diseases); assessment of visual acuity; fundus examination; evaluation of the extent of vision loss via a visual field test (Goldmann visual field); evaluation of retinal activity using ffERG; retina assessment using imaging techniques, namely OCT and FAF [6, 11, 39]. Although ERG and Goldmann perimetry continue to be crucial functional tests, retina imaging with OCT and FAF have contributed significantly to our understanding of the retinal structure and degeneration and represent essential diagnostic tools. A newer tool, the full-field stimulus threshold (FST), an alternative test for assessing retinal function in patients where ERG is not able to be performed or where ERG responses are undetectable, is also making its way into clinical practice as a valuable tool for further diagnostic investigations, such as the evaluation of treatment effects [40], and is now widely available in centers that manage IRDs [9].

An ongoing exchange between child neuropsychiatrists, ophthalmologists, pediatricians, and geneticists is crucial to establish and better define the different steps of diagnosis jointly. Additional investigations such as brain magnetic resonance imaging (MRI), electroencephalogram (EEG), metabolic examinations, abdomen ultrasound, in particular in the presence of neurological and systemic associated symptoms, and genetic analysis may also be considered in order to differentiate non-syndromic from syndromic forms, such as ciliopathies (e.g., Joubert’s syndrome) [41–43], and peroxisomal and mitochondrial diseases [44, 45]. Neurological symptoms such as hypotonia, neuromotor deficit, ataxia, epilepsy, and auditory, skeletal, renal and hepatic abnormalities can be further evaluated to confirm a diagnosis.

### Clinical investigations

- Perimetry

In the early stages of IRD photopic visual field may appear complete. However, small relative scotomata may be located in the region between 20° and 40° from the fixation point and will fuse progressively, with the appearance of a relatively dense annular scotoma in the area between 20° to 45° from the fixation point [25]. During the course of the disease, the annular scotoma extends toward both the center and the periphery. Eventually, tunnel vision will develop, with a central field between 5° and 10° around the fixation point. Kinetic perimetry is most suitable for the assessment of peripheral visual field loss.

(b) Electroretinography

The ERG technique measures the electrical activity of retinal photoreceptor cells and provides information on the activity of rod and cone cells in response to light. Decreased electrical activity indicates a loss of photoreceptor function.

The use of ffERG for adults and, when necessary, using abbreviated protocols in children, represents a fundamental tool for diagnosing IRDs supported by the guidelines of the International Society for Clinical Electrophysiology of Vision (ISCEV) (http://www.iscev.org/standards). It reveals the characteristic loss of photoreceptor function, which occurs principally among rod photoreceptors rather than cones in the early stages of the disease [6]. Indeed, by comparing the loss of rod- and cone-mediated responses, one can distinguish between a disease involving primary rod photoreceptors and secondarily cones, like typical RP and congenital stationary night blindness (CSNB), from a primary dysfunction and degeneration of both rods and cones, as in cone-rod dystrophies, and from a selective dysfunction/degeneration of cones with no or minimal involvement of rods, as in cone dystrophies, achromatopsia, and blue cone monochromatism.

ERG abnormalities precede occur in the early stages, preceding night blindness symptoms and fundus abnormalities in RP. An undetectable or at least severely reduced ERG recording is typical in LCA [6, 31, 32]. ERG is also important for diagnosing atypical forms without pigments from congenital hemeralopia. As the disease progresses, ffERG may become non-recordable despite a residual visual field, in which case FST provides a fast test while circumventing the fixation requirement. Alternatively, a multifocal ERG (mfERG) may still be able to elicit responses and help monitor disease progression. Delayed responses in the mfERG may predict visual field loss in a retina that otherwise appears to be healthy [6].

(c) Optical coherence tomography

Optical coherence tomography allows the visualization of the thinning or disappearance of retinal layers, including the photoreceptor layer of the posterior pole. The use of OCT in IRDs has been well described, particularly in RP, where histopathological changes are first shown by a shortening of the photoreceptor outer segments [46], with confirmation seen on SD-OCT imaging, that shows disorganization of the outer retinal layers, first at the interdigitation zone, then the ellipsoid zone (EZ), and finally occurring at the external limiting membrane [47, 48]. In particular, in sector RP, OCT shows the differences between the affected and non-affected retinal areas [49].

Disruption of the EZ zone is associated with a worse prognosis in RP. More specifically, the restoration of normal vision in terms of visual acuity can be expected in patients with an EZ width of approximately 600 μm [50]. As RP progresses, there is a thinning of the outer segments accompanied by a decrease in the thickness of the outer nuclear layer (ONL), the location of photoreceptor cell nuclei. In the late stages of RP, there is a complete loss of both the outer segment and the ONL [6, 51].

In patients with RP, hyperreflective foci (HRF) are frequently found in the outer layers of the central macula, at the edge of the atrophic outer retina, and in the choroid, associated with a decrease in macular thickness accompanied by visual impairment. The concomitant presence of HRF could lead to more profound visual impairment and marked visual decline during monitoring [52]. OCT imaging also has a role in diagnosing other macular abnormalities, such as macular edema and atrophic changes, that are present in up to 50% of patients with RP [47, 48, 53].

Changes in superficial and deep vascular density and enlarged foveal avascular zone have been demonstrated with OCT angiography (OCT-A) [54], which also allows the study of the choroidal tissue, which it is not possible to evaluate accurately using other imaging techniques: choroidal thickness is significantly lower in the RP in comparison normal subjects [55].

In some LCA genotypes, OCT studies may initially show relatively normal outer retinal morphology, whereas normal or subnormal retinal and ONL thickness may be observed in other patients; viable photoreceptor the presence of viable photoreceptors appears to persist until relatively later in the disease process [31, 32].

(d) Fundus autofluorescence imaging

Fundus autofluorescence is an essential diagnostic tool for diagnosing and monitoring IRDs that can detect otherwise-undetectable disruptions in RPE metabolism.

**Fig. 1:**
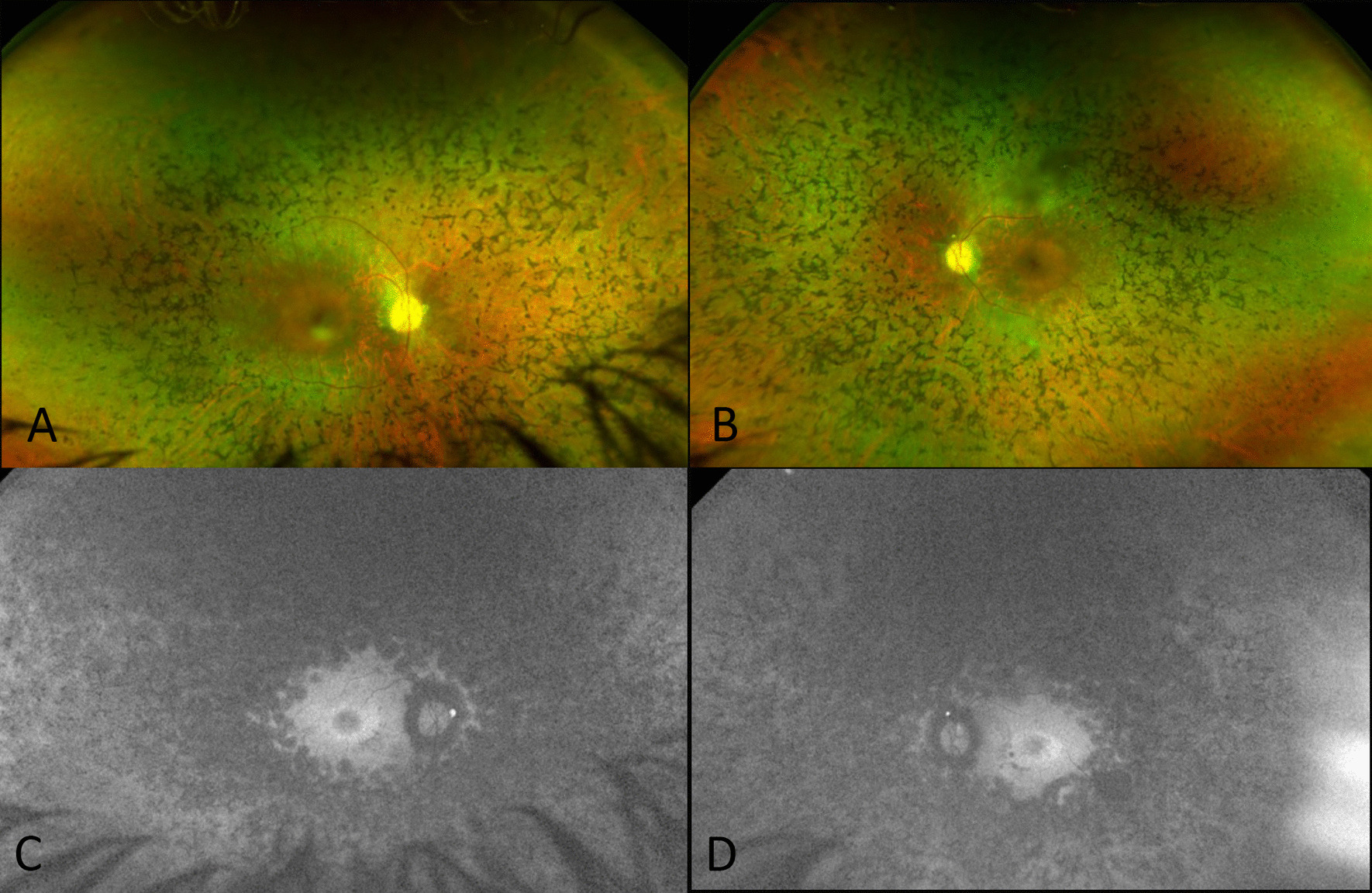
Wide-field fundus photographs (A, B) and autofluorescence imaging (C, D) of the right and left eye of a patient with retinitis pigmentosa. Wide-field fundus photographs showing widespread retinal dystrophy with pigment clumping, narrowing of retinal vessels and pale optic disc. Fundus autofluorescence showing hyperautofluorescence ring at the macular region, widespread hypoautofluorescence abnormalities in mid and far periphery and small optic disc drusen in both eyes

**Fig. 2:**
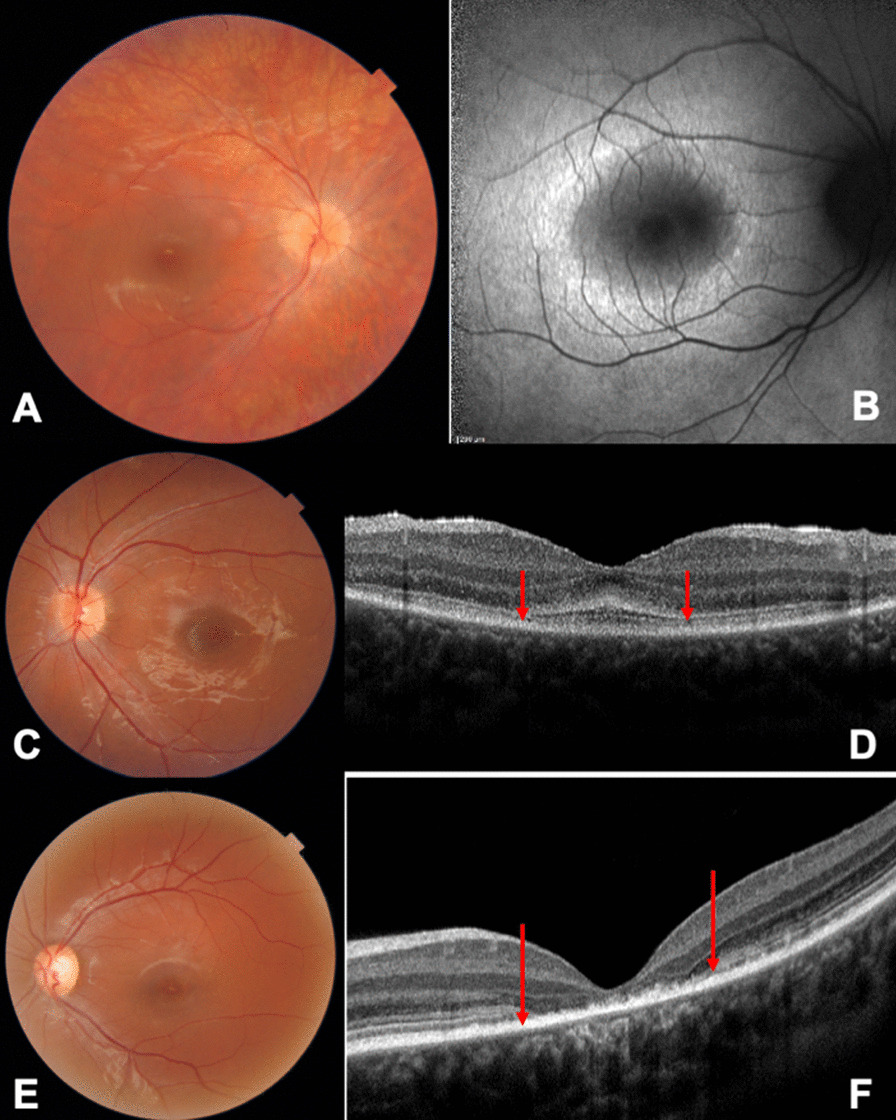
Fundus photography and optical coherence tomography (OCT)/ fundus autofluorescence (FAF) of pediatric patients showing silent fundus images, but evocative OCT and FAF picture of early-onset inherited retinal dystrophy (IRD). **A, B**: Fundus image and FAF of early onset IRD in an 8-year-old child with rod-cone dystrophy. Note the hyperautofluorescence ring at the posterior pole in FAF. **C, D**: Fundus image and OCT scan of a 5-year-old child with early-stage ABCA4 maculopathy. Note the thickening of the outer limiting membrane (red arrows). **E, F**: Fundus image of a 10-year-old child (brother of C, D) showing silent fundus image but clear altered ellipsoid zone and macular atrophy

Lipofuscin in the RPE is the principal source of the autofluorescent signal using short-wavelength (SW)-FAF (blue or green light). Hyperautofluorescence is correlated with metabolic dysfunction of the retina, and hypoautofluorescence is correlated with atrophy of the RPE [56–58] (Fig. 1). An abnormal foveal ring or curvilinear arc of increased autofluorescence not visible on ophthalmoscopy is present in 50–60% of RP patients [48, 57]. Such an hyperautofluorescent ring can represent a transition zone between normal and abnormal retinal function, with relatively normal function within the ring and absent function outside it. More specifically, the hyperautofluorescent ring denotes an area of outer segment dysgenesis and lipofuscin production, with progressive retinal thinning usually associated with loss of the ellipsoid zone at (or close to) the inner edge of the ring [58] (Fig. 2). The diameter of the ring grows smaller over time, in parallel with the reduction of the visual field [6, 48]. Recently, ultra-wide field (UWF) imaging allowed the evaluation of the FAF abnormalities of the retinal periphery and revealed that the hypoautofluorescent areas at the FAF examination co-localize with the visual field scotomata seen on Goldmann perimetry in patients with RP [59, 60]. Other autofluorescence patterns, such as central hyperautofluorescence, triple hyperautofluorescent rings and paravascular hyperautofluorescent alterations, can be observed in addition to the hyperfluorescent ring [57, 60, 61]. Certain retinal degenerations may also exhibit an overall low FAF signal due to an enzymatic dysfunction of the visual cycle, such as *RPE65* Leber congenital amaurosis and fundus albipunctatus [58, 62,58, 62].

### Molecular diagnosis

The goal of genetic testing is to achieve a molecular diagnosis that allows a proper genotype–phenotype correlation while confirming the clinical diagnosis and distinguishing the IRD from other forms of retinal disease. In addition to providing a more accurate prognosis of the likely visual outcome, an accurate molecular diagnosis facilitates developing appropriate follow-up and rehabilitation, establishing eligibility for available gene therapies, and estimating recurrence risk for future offspring and other family members [2, 3, 8, 9]. The proband’s parents should also be included in genetic testing when available. A molecular diagnosis also represents the basis for planning effective genetic counseling for affected families.

Recent advances in molecular genetic techniques, such as targeted multi-gene NGS sequencing using large gene panels, have greatly aided and simplified molecular diagnosis. However, the considerable variability and overlap of disease presentation in patients with IRDs still present challenges in achieving an accurate molecular diagnosis. Genetic laboratories must maintain the high level of competence essential to provide accurate genotypic characterization.

Since its introduction, NGS-based genetic testing has revolutionized the diagnosis of IRDs by facilitating the identification of causative genes responsible for the diseases [10, 12, 13, 16]. It is now possible to identify the pathogenic variants in up to 70%, or even more, of cases. As of October 2022, over 280 genes causing IRD had been identified [24]. The identified genes often encode key molecules of retinal phototransduction and the visual cycle.

In the past years, a problem in identifying emerging pathogenic variants was the lack of consensus between laboratories. It was estimated that laboratories disagreed in interpreting a variant in 17% of cases [63]. To overcome this problem, guidelines for interpreting sequence variants issued by the American College of Medical Genetics and Genomics and the Association of Molecular Pathology [64] provide criteria for the interpretation of the variant pathogenicity (for example, allele frequency > 5%, in general, suggests benignity) [64]. However, some exceptions exist (for example, mild *ABCA4* variants with higher allele frequency values have been identified in Stargardt disease) [65]. Several computational predictive programs have been developed to aid in interpreting sequence variants [64, 66, 67]. They provide algorithms that help to determine the effect of a sequence variant at the nucleotide and amino acid level, allowing the effect of the genetic variant on the primary and alternative gene transcripts to be determined, along with other genomic elements, including information on the potential impact of the variant on the protein function.

Variants that fail to meet the criteria for interpretation are classified as “of uncertain significance” (VUS) [66]. The resolution of VUS cases requires segregation analysis and collaboration between centers. Crosschecking information gathered from genetic testing across different databases (Human Genome Mutation Database, gnomAD, VarSome) is highly recommended. Variant classification is dynamic, and classified variants need periodic re-evaluation [68, 69].

There remain limitations and unresolved issues relating to genetic testing for IRDs. For about 30–40% of patients with an IRD, the genetic cause remains unclear [70]. The reasons for cases of genetically unsolved IRD include the presence of mutations undetectable/uninterpretable by NGS. For example, highly repetitive sequences that require a dedicated Sanger approach, deep intronic sequences that affect splicing mechanisms, small deletions/duplications that in some cases can also have an impact on topological-associated domains in the genome, VUS, unexpected phenotype-genotype correlations, and novel IRD genes [8, 12, 71]. The interpretation of VUS is challenging [72, 73] and will continue to be refined through further research.

### Genetic counseling

Genetic counseling allows the individual and families to understand the condition and the potential risk for offspring, aids informed reproductive decisions and helps explain the implications of the diagnosis on visual prognosis and treatment options. While counseling after genetic testing is essential to explain and interpret the results obtained, patients and their families should also receive counseling before testing so that the advantages and potential disadvantages of a genetic test are understood in advance. Ideally, counseling by at least a medical geneticist with in-depth knowledge of the genetic aspects of IRDs, supported where possible by a genetic counselor, forms an effective two-way communication process that helps those involved to understand the information, make appropriate choices about the course of action that needs to be followed and facilitates coming to terms with the condition.

As patients’ expectations and patient-reported outcomes are increasingly being considered in clinical trial design in rare diseases [74, 75], it is important to inform patients clearly, understand their expectations, and be available for and ready to answer their questions [74, 76]. A genetic test usually raises many questions in the minds of a child’s parents. Without creating unrealistic expectations, it should be explained that the genetic test is part of the therapeutic process and may reveal the eligibility for available and/or future treatments. The time taken to provide effective genetic counseling is rewarded by a greater sense of control over the condition for the patient and their family.

### Disease management

**Fig. 3:**
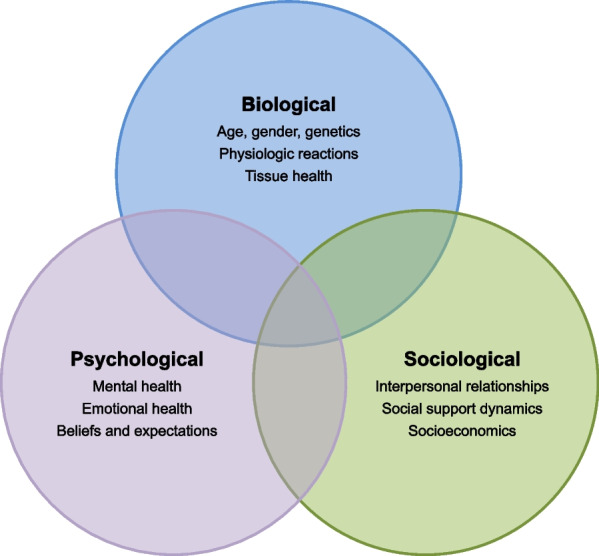
The biopsychosocial model provides a unitary and global approach to the person and represents a good platform for an interdisciplinary approach between the various professions involved in managing patients with inherited retinal diseases

The multidisciplinary (or interdisciplinary) approach is based on the biopsychosocial model (Fig. 3**)**, which uses biological, psychological, and sociological factors and their complex interrelationships to understand the psychophysical health of the patient and guide the choice of therapeutic intervention [77, 78]. It provides a unitary and global approach to the person and represents a good platform for an interdisciplinary approach between the various professions involved. IRDs require such an approach from the developmental age and throughout youth and young adulthood. The professionals involved may differ at different stages of the subject’s age, and not all participate at the same time in the process of care. In reality, IRDs are chronic conditions in which the needs of the person, family members, and caregivers change across the different phases of life and with the progression of the disease. Caregiving of individuals with IRDs is long-term and needed for the person's entire lifetime.

**Fig. 4:**
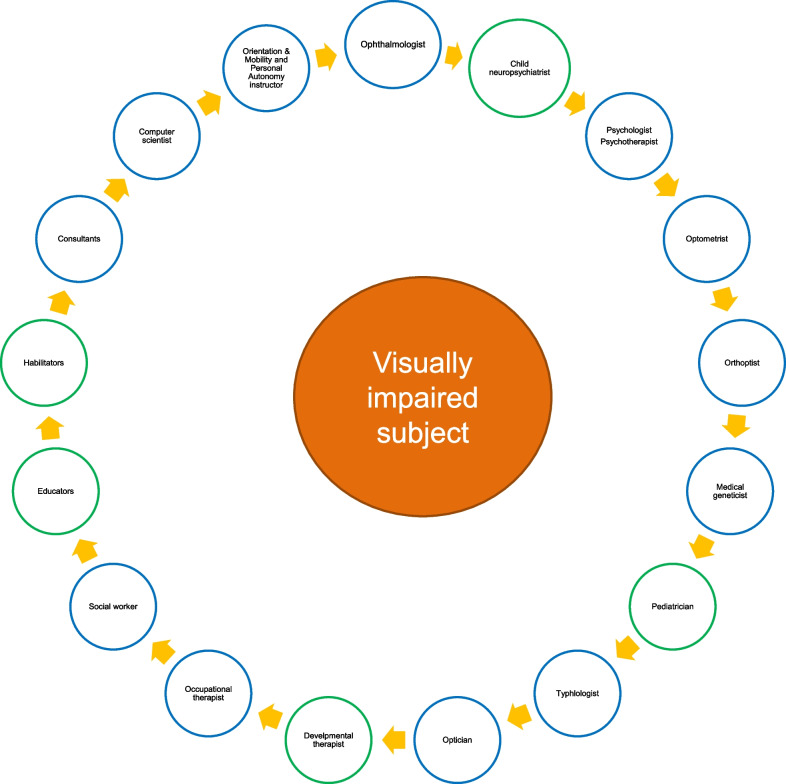
The patient-centered multidisciplinary team approach to vision rehabilitation in visually impaired children and young/young-adult persons with inherited retinal dystrophies. The green circles identify the professionals dedicated exclusively to the subject in the developmental age

The individual must be placed at the center of an ideal circle around which the various professionals revolve (Fig. 4) while, at the same time, the person participates in their own course of treatment. It is important that emotional support is provided to the child and caregivers at diagnosis and that a coordinated approach is followed to guide the family through all stages of care and habilitation or rehabilitation and to facilitate the registration process. This is designed to ensure appropriate benefits and support are issued. In Italy, this role may be performed by genetic counselors and low vision specialists, although specific Eye Clinic Liaison Officers may form part of the multidisciplinary team in some countries.

This is person-centered care, a method of approaching illness and treatment recommended by the World Health Organization [79], which sees health as an interplay of physical, mental, and social well-being and not merely the absence of disease or infirmity. This concept of health correlates closely with perceived quality of life. In all chronic conditions, the goal of treatment is to contain the damage, slow, if possible, the progression of the disease, and improve the person’s well-being.

Caring for children with IRDs demands close collaboration among an interdisciplinary team that may include pediatricians, optometrists, ophthalmologists, orthoptists, low vision specialists, medical geneticists, child neuropsychiatrists, psychologists, developmental therapists, typhlologists, educators, occupational therapists, orientation and mobility specialists, habilitators (for the very young) and rehabilitators (for older children). The professionals involved should have in-depth knowledge of the genetic aspects of IRDs and the management of vision disorders beyond their knowledge of syndromic disorders. This approach, recommended for diagnosis, follow-up, and rehabilitation management, is illustrated in Fig. 4.

Particularly in the first years of life, the functional impact of vision impairment can manifest itself not only with visual clinical signs such as nystagmus, low vision, and abnormal behavior to light but also with clinical signs regarding development such as reduced emotional facial expression, motor initiative, and interactive proactivity, stereotypies, lack of interest for the environment. In older children, it is important to look also for behavioral problems, reading difficulties, reduced mobility, navigation difficulties (e.g., in different light conditions of the environment), a cautious attitude in orienting themselves in new spaces or interacting with peers that could be related to the vision impairment.

Vision plays a key role in a child’s overall development. From this perspective, it is clear that congenital and/or early-onset visual disorders could jeopardize the structuring of crucial functions: communicative-relational (from the very earliest stages of the mother–child bonding process), cognitive-neuropsychological, motor (postural control, action) [80] and spatial. The development of the visually-impaired child has peculiarities and signs of risk that are important to recognize in order to set up good management, and it is also very important to know not only how the eye functions but also how the person functions [81]. We need to consider tasks such as reading, mobility, face recognition, social context, and quality of life. These reasons reinforce the need to involve the kind of broadly-skilled multidisciplinary or interdisciplinary team outlined above, starting at diagnosis and continuing throughout the habilitation and rehabilitation phases.

Rehabilitation or habilitation, as appropriate according to developmental age, often begins from the time of diagnosis, and caring also extends to the entire family from this time. Ideally, the intervention will be: (a) interdisciplinary; (b) multisensory, with activities that encourage the integration of different perceptual information coming from the environment; (c) individualized, that is based on the visual and developmental profile of the child and his overall clinical picture; (d) multidimensional, because its rehabilitation goals rely on collaboration between professionals and caregivers across the different contexts of life and the entailing activities also related to home, school and social settings [82]. Two broad categories of intervention can be considered in determining rehabilitation objectives; those targeting the development of visual and alternative sensory skills (e.g., touch, hearing) and their functional use and those designed to prevent or reduce a possible negative impact of the visual impairment on overall development [82]. Visual support, such as low vision aids, computer screen readers, audiobooks, and tablet computer-based assistive technologies such as optical character recognition, screen magnifiers, or text-to-speech conversion, can help children compensate for diminishing visual function [82].

Parents are offered psychological support to assist their adjustment to and coping with the diagnosis while sustaining the parent child-relationship and promoting understanding of the child’s needs and means of expression. As the patient grows, psychological care is planned for them, either individually or in groups. Ultimately, such an intervention aims to encourage independence in the child and improve the quality of life, social participation, and integration.

In the young, young-adult subject, unlike the typical management of developmental age, it is possible to identify two multidisciplinary teams that intervene at different times: the “diagnostic team” and the “rehabilitation or habilitation team”. The “diagnostic team” consists of the ophthalmologist, the medical geneticist, and the psychologist (Fig. 4). Where present in the team, the psychologist is a fundamental figure in supporting communication at the diagnosis and ensuring individual support is available if necessary [83].

Later, ocular disease progression requires starting with vision rehabilitation [84]. In this phase of the treatment process, the team consists of the ophthalmologist, the psychologist, the orthoptist, the orientation and mobility and personal autonomy instructor, the typhlologist, and the computer scientist.

The habilitation or rehabilitation project is timed and individualized to the individual’s needs, requests, and clinical condition [85], considering the patient’s emotional state and readiness to deal with the demands of the rehabilitation process. As a key team member, the psychologist supports not only the person, family members, and caregivers, but provides emotional support to other team members to minimize potential burnout.

The overall objective of vision rehabilitation is to assist the subject to find the best strategies to take advantage of their residual vision, promoting the integration of the “new self”, which translates into an improvement in perceived well-being and quality of life.

## Summary

- The effective management of inherited retinal dystrophies (IRDs) requires close collaboration between members of a multidisciplinary, or ideally an interdisciplinary, team of experts knowledgeable about IRDs, including suitably experienced professionals from the fields of ophthalmology, neuropsychiatry, psychology, neurology, genetics, orthoptics, developmental therapy, typhlology, occupational therapy, otolaryngology, and/ orientation and mobility specialties.
- As IRDs usually present at an early age, clinicians need to be aware of the clinical signs suggesting visual impairment and follow up with timely diagnoses to facilitate appropriate therapeutic or rehabilitation interventions to minimize vision loss.
- The highly heterogeneous nature and overlapping of manifestations among the different IRDs make reaching a definitive diagnosis difficult. Accurate clinical diagnosis is a prerequisite for genetic testing and requires a range of objective and subjective assessment measures to ensure appropriate treatment and optimization of outcomes.
- The diagnostic process should involve taking an accurate medical history, consideration of family history, fundus examination, evaluation of visual acuity, the extent of vision loss via a visual field test (Goldmann visual field), retinal activity (full-field electroretinogram), and retina assessment using imaging using optical coherence tomography and fundus autofluorescence. Full-field light sensitivity testing is also a valuable tool for further diagnostic investigation.
- Establishing the genotype of IRDs is increasingly considered essential in the diagnostic workup, particularly since the introduction of voretigene neparvovec as an effective gene therapy for treating adult and pediatric patients with vision loss due to IRDs caused by confirmed biallelic mutations in the *RPE65* gene.
- The complex process of determining IRD-causing gene variants to reach a molecular diagnosis takes advantage of improvements in genome sequencing techniques, notably next-generation sequencing.
- Patients and their families must receive counseling before and after genetic testing; effective communication is essential, and patient and parents’ expectations must be acknowledged and discussed.
- Psychological support for patients and caregivers is important at all stages of diagnosis, care, and rehabilitation, and the child neuropsychiatrist plays an essential role in managing IRDs.
- Comprehensive multidisciplinary care plans for patients with IRD have been developed to provide streamlined and patient-friendly access to clinical and molecular diagnoses, accelerate access to available therapeutic options and support, and for referral to standardized clinical care and rehabilitation.
- Despite the solid collective effort in the field of advanced diagnosis and novel therapies for IRDs, further knowledge is required about the natural history of the diseases that could be helpful to better evaluate treatment effects, particularly in these slowly progressive diseases. Given the rarity of each form of IRD, this usually needs the development of multicentric studies designed with appropriate and robust protocols.

## Conclusions

Timely and accurate diagnosis and appropriate interdisciplinary management involving ophthalmologists, geneticists, and other medical specialists have important implications for the overall development and life-long visual function and quality of life of children with IRDs.

## Data Availability

Not applicable.

## Abbreviations

CME: Cystoid macular edema
EEG: Electroencephalogram
EOSRD: Early-onset severe retinal dystrophy
ERG: Electroretinogram
EZ: Ellipsoid zone
FAF: Fundus autofluorescence
ffERG: Full-field electroretinogram
FST: Full-field stimulus threshold
HRF: Hyperreflective foci
ISCEV: International Society for Clinical Electrophysiology of Vision
IRD: Inherited retinal dystrophy
LCA: Leber congenital amaurosis
mfERG: Multifocal electroretinogram
MRI: Magnetic resonance imaging
NGS: Next-generation sequencing
OCT: Optical coherence tomography
OCT-A: OCT angiography
ONL: Outer nuclear layer
RP: Retinitis pigmentosa
RPE: Retinal pigment epithelium
SD-OCT: Spectral domain OCT
SECORD: Severe early childhood onset retinal dystrophy
UWF: Ultra-wide field
VUS: Variant of uncertain significance
